# Efficacy and safety of pharmacotherapies for smoking cessation in anxiety disorders: Subgroup analysis of the randomized, active‐ and placebo‐controlled EAGLES trial

**DOI:** 10.1002/da.22982

**Published:** 2019-12-18

**Authors:** Catherine R. Ayers, Jaimee L. Heffner, Cristina Russ, David Lawrence, Thomas McRae, A. Eden Evins, Robert M. Anthenelli

**Affiliations:** ^1^ Department of Psychiatry University of California San Diego California; ^2^ Public Health Sciences Division Fred Hutchinson Cancer Research Center Seattle Washington; ^3^ Global Product Development Pfizer New York New York; ^4^ Center for Addiction Medicine Massachusetts General Hospital and Harvard Medical School Boston Massachusetts

**Keywords:** anxiety/anxiety disorders, GAD/generalized anxiety disorder, panic disorder, PTSD/posttraumatic stress disorder, smoking

## Abstract

**Background:**

Smoking rates are high in adults with anxiety disorders (ADs), yet little is known about the safety and efficacy of smoking‐cessation pharmacotherapies in this group.

**Methods:**

Post hoc analyses in 712 smokers with AD (posttraumatic stress disorder [PTSD], *n* = 192; generalized anxiety disorder [GAD], *n* = 243; panic disorder [PD], *n* = 277) and in a nonpsychiatric cohort (NPC; *n* = 4,028). Participants were randomly assigned to varenicline, bupropion, nicotine‐replacement therapy (NRT), or placebo plus weekly smoking‐cessation counseling for 12 weeks, with 12 weeks follow‐up. General linear models were used to test the effects of treatment group, cohort, and their interaction on neuropsychiatric adverse events (NPSAEs), and continuous abstinence weeks 9–12 (treatment) and 9–24 (follow‐up).

**Results:**

NPSAE incidence for PTSD (6.9%), GAD (5.4%), and PD (6.2%) was higher versus NPC (2.1%), regardless of treatment. Across all treatments, smokers with PTSD (odds ratio [OR] = 0.58), GAD (OR = 0.72), and PD (OR = 0.53) had lower continuous abstinence rates weeks 9–12 (CAR9–12) versus NPC. Varenicline demonstrated superior efficacy to placebo in smokers with GAD and PD, respectively (OR = 4.53; 95% confidence interval [CI] = 1.20–17.10; and OR = 8.49; 95% CI = 1.57–45.78); NRT was superior to placebo in smokers with PD (OR = 7.42; 95% CI = 1.37–40.35). While there was no statistically significant effect of any treatment on CAR9–12 for smokers with PTSD, varenicline improved 7‐day point prevalence abstinence at end of treatment in this subcohort.

**Conclusion:**

Individuals with ADs were more likely than those without psychiatric illness to experience moderate to severe NPSAEs during smoking‐cessation attempts, regardless of treatment. While the study was not powered to evaluate abstinence outcomes with these subgroups of smokers with ADs, varenicline provided significant benefit for cessation in those with GAD and PD, while NRT provided significant benefit for those with PD.

## 1 INTRODUCTION

Individuals with anxiety disorders (ADs) smoke tobacco at rates 2–3 fold higher than those without mental health conditions (Cougle, Zvolensky, Fitch, & Sachs‐Ericsson, 2010; Lasser et al., 2000; McCabe et al., 2004). Like other smokers with mental health conditions (Evins, Cather, & Laffer, 2015), smokers with AD typically smoke more heavily (Kelly, Jensen, & Sofuoglu, 2015aa), are more severely nicotine‐dependent (Okoli, Otachi, Manuel, & Woods, 2018), and experience earlier and more severe nicotine‐withdrawal symptoms (Piper, Cook, Schlam, Jorenby, & Baker, 2011) than smokers without mental health conditions, making quitting smoking more challenging (Kelly et al., 2015bb). Despite this high rate of co‐occurrence and greater difficulty quitting, relatively few randomized controlled trials (RCTs) have evaluated the safety and efficacy of the front‐line smoking cessation pharmacotherapies in smokers with AD. Moreover, most RCTs conducted to date of smoking cessation interventions for smokers with AD included no placebo control or comparison group of smokers without mental health conditions, focused on one AD condition at a time, and tested only one of the US Food and Drug Administration (FDA)‐approved medications in that AD subcohort. Thus, there are insufficient data to draw comparisons across different diagnostic subgroups of smokers with AD, control smokers, and smoking cessation medications.

A notable exception was the retrospective analysis of a placebo‐controlled RCT of nicotine‐replacement therapy (NRT) and bupropion in a subcohort of smokers who endorsed prior panic attacks (not necessarily meeting criteria for panic disorder [PD]), social phobia, or generalized anxiety disorder (GAD; Piper et al., 2011). Smokers with AD in this study had lower abstinence rates than smokers without mental health conditions and appeared to derive no added benefit from bupropion, NRT, or their combination over placebo plus counseling. While there was some specificity between the subtype of ADs, the results were more similar than different. To our knowledge, no placebo‐controlled RCTs have examined the efficacy of varenicline in smokers with ADs, most likely reflecting a reluctance to prescribe this effective medication to individuals with ADs because of concerns raised about its neuropsychiatric safety (Anthenelli et al., 2016). For example, a retrospective chart review of 78 smokers with posttraumatic stress disorder (PTSD) found an increased number of mental health encounters while subjects were taking varenicline compared with a pretreatment baseline period (Campbell & Anderson, 2010). Such findings raised concerns about the use of varenicline in smokers with PTSD, yet without the inclusion of placebo control, the mental health encounters cannot be attributed to medication effects. Further, given the historical concerns about varenicline and subsequent restrictions placed on its use by several federal agencies (Federal Aviation Administration, 2008; US Department of Defence, 2008), including the Department of Veterans Affairs Health System for patients with mental health conditions (US Department of Veterans Affairs et al., 2008), examining varenicline in the largest randomized trial to date will provide critical safety information to guide policies and practices.

Given the paucity of safety and efficacy data from methodologically rigorous, placebo‐controlled RCTs of pharmacotherapeutic cessation aids, clinicians have had little guidance on how to advise patients with specific AD diagnoses about which cessation treatment might work best for them. It is also not clear whether cessation obstacles equally cut across this heterogeneous group of mental health conditions (i.e., PTSD, GAD, and PD with/without agoraphobia) that form the broader AD category.

The multinational EAGLES (Evaluating Adverse Events in a Global Smoking Cessation Study; Anthenelli et al., 2016), which compared the non‐nicotine medications varenicline and bupropion with an active comparator (transdermal nicotine patch, NRT) and placebo in smokers with/without mental health conditions, provides a unique opportunity to address these knowledge gaps. With approximately 20% (*n* = 787) of the EAGLES psychiatric cohort having a primary AD diagnosis, it is the largest sample of smokers with AD ever enrolled in a placebo‐ and active‐controlled RCT for smoking cessation treatments, and the first to compare all three first‐line smoking cessation aids head‐to‐head in such smokers. In this planned secondary analysis we compare the safety and efficacy of varenicline, bupropion, NRT, and placebo across AD subcohorts (PTSD, GAD, and PD), and compare clinical characteristics and rates of clinically significant neuropsychiatric adverse events (NPSAEs) and cessation in smokers with AD versus a cohort of smokers without psychiatric disorders.

## 2 MATERIALS AND METHODS

### 2.1 Study design

EAGLES was a multinational RCT (http://ClinicalTrials.gov identifier NCT01456936) conducted at the request of the FDA and European Medicines Agency. It was an active treatment‐ (transdermal nicotine patch, NRT) and placebo‐controlled trial of varenicline and bupropion for 12 weeks, with a 12‐week nontreatment follow‐up assessment. Institutional review boards or ethics committees at participating institutions approved the study procedures. The study adhered to the Declaration of Helsinki and the International Conference on Harmonization Good Clinical Practice Guidelines. All patients signed informed consent. The main outcome paper provides details of the study design (Anthenelli et al., 2016). Here we present a post hoc subgroup analysis of the EAGLES safety and efficacy outcomes identical to the parent trial.

### 2.2 Participants

Eligible participants were adults aged 18–75 years who smoked an average of ≥10 cigarettes/day with an exhaled carbon monoxide (CO) >10 parts per million (ppm) at screening and wanted to stop smoking. AD subcohort participants met diagnostic criteria based on the *Diagnostic and Statistical Manual of Mental Disorders, Fourth Edition, Text Revision* (DSM‐IV‐TR) for primary PD (with/without agoraphobia; *n* = 277), PTSD (*n* = 192), or GAD (*n* = 243), confirmed by the Structured Clinical Interview for DSM‐IV‐TR Axis I Disorders (SCID‐I; American Psychiatric Association, 2000; First, Gibbon, Spitzer, Williams, & Benjamin, 1997). Smokers with a primary diagnosis of obsessive‐compulsive disorder (*n* = 27) and social phobia disorder (*n* = 48) were also enrolled in the parent trial but not included in the present analysis because of the small sample size (Figure S1). Participants could have a comorbid psychiatric diagnosis, provided it did not prevent the participant from complying with study requirements. If the comorbid diagnosis was a substance‐use disorder, the participant was required to be in sustained full remission to be eligible. AD participants must have been considered psychiatrically stable as evidenced by no exacerbations of their psychiatric condition in the prior 6 months and if on pharmacotherapy, to have been on a stable dose for ≥3 months. They could not be considered at high risk of self‐injurious or suicidal behavior. Nonpsychiatric cohort (NPC) participants were confirmed via SCID to have no major psychiatric disorders (*n* = 4,028).

### 2.3 Randomization and treatment

In EAGLES, participants were stratified into one of four psychiatric subcohorts that included those individuals with AD, or the NPC, while also stratifying into one of four geographic regions. Subsequently, participants were randomized to study treatment in a 1:1:1:1 ratio using a computer‐generated schedule. They were not randomized based on their specific AD diagnosis but stratified by whether they had any AD. Participants received either varenicline 1 mg twice daily, bupropion 150 mg twice daily, transdermal nicotine patch (NRT) 21 mg/day with taper, or placebo for 12 weeks. Treatment was administered in a double‐blind, triple‐dummy fashion (i.e., participants received one active and two placebo medications, or three placebo medications), and all assessments were blinded throughout the study. All participants received brief weekly smoking cessation counseling (e.g., managing withdrawal and cravings) that lasted <10 min and was based on US Agency for Healthcare Research and Quality Guidelines (Fiore et al., 2008). No routine mental health counseling was provided for mental health issues, including ADs.

### 2.4 Outcomes

#### 2.4.1 Safety

The primary safety outcome was the occurrence of any moderate to severe NPSAEs, which were operationally defined with guidance from the FDA (Anthenelli et al., 2016). Criteria for the primary neuropsychiatric endpoint required the four categories of NPSAEs (anxiety, depression, feeling abnormal, or hostility) most commonly associated with nicotine withdrawal to be rated as severe (i.e., significantly interfering with functioning). The other 12 categories (agitation, aggression, delusions, hallucinations, homicidal ideation, mania, panic, paranoia, psychosis, suicidal ideation, suicidal behavior, or completed suicide) were included in the primary composite safety endpoint if they were rated as either moderate (i.e., some interference with functioning) or severe in intensity. Secondary safety endpoints included the incidence of each of the individual components and the subset of NPSAEs that were rated severe. We also examined general AEs of any intensity (mild, moderate, or severe) including those in the neuropsychiatric domain.

#### 2.4.2 Efficacy

The primary efficacy outcome measure was continuous abstinence rates weeks 9–12 (CAR9–12) and secondary efficacy was CARs weeks 9–24 (CAR9–24). Continuous abstinence was defined by self‐report of no smoking (even a puff), with an expired CO concentration of ≤10 ppm (Benowitz et al., 2002). Seven‐day point prevalence abstinence (PPA) rates at week 12 (end of treatment) and week 24 (end of follow‐up) were also evaluated as prespecified secondary endpoints.

### 2.5 Assessments

Psychiatric diagnoses were evaluated using SCID‐I and ‐II (First et al., 1997; First, Spitzer, Gibbon, & Williams, 2002). The severity of cigarette dependence was assessed at baseline with the Fagerström Test for Cigarette Dependence (FTCD; Fagerström, 2012). Anxiety and depression symptoms were assessed at baseline and serially over time using the Hospital Anxiety and Depression Scale (HADS; Zigmond & Snaith, 1983). The Buss‐Perry Aggression Questionnaire (Buss & Perry, 1992) was used to assess trait aggression at baseline. NPSAEs were assessed with open‐ended questions, direct observation, and a 25‐item semi‐structured Neuropsychiatric Adverse Events Interview (Anthenelli et al., 2016; Anthenelli et al., 2013) done at each study visit which solicited psychiatric complaints across the 16 domains that comprised the primary safety outcome. Further, NPSAEs were evaluated through reports from family members or responses on the HADS and Columbia Suicide Severity Rating Scale (Posner et al., 2011).

### 2.6 Statistical analysis

Stepwise logistic regression (using a 5% level for entry/stay) was used to model the safety and efficacy endpoints of interest. Design terms for treatment, diagnostic subcohort, their interaction, and region (US or non‐US), had forced inclusion (efficacy included cohort by region interaction as well). Several baseline characteristics, both numerical and categorical, formed the set of candidate covariate terms. Safety analyses included all participants who took ≥1 dose of randomized treatment. Efficacy analyses of CARs included all randomized participants. Consistent with the Russell standard (West, Hajek, Stead, & Stapleton, 2005), participants with missing smoking data were considered nonabstinent. The final model summaries for NPSAEs, CA9–12, CA9–24, and PPA at weeks 12 and 24 are provided in Table ST1.

## 3 RESULTS

### 3.1 Baseline characteristics

There were 712 participants in the AD subcohort and 4,028 in the NPC. Baseline demographic, smoking, and psychiatric characteristics are displayed in Table 1 and demonstrate both AD subcohort and AD diagnostic subcohorts effects. For example, smokers with AD were more likely to be female, from the USA and of white race than NPC smokers. Smokers with PD had higher FTCD scores than NPC smokers. Smokers with AD had more psychiatric and substance‐use comorbidity and greater lifetime suicidal ideation and behavior. In particular, at least descriptively, smokers with PTSD had higher rates of comorbid alcohol‐use disorder history, other psychiatric disorders, prior suicidal ideation and/or behavior, and were less likely to be on psychotropic medication than smokers with GAD and PD. As expected, the HADS anxiety score was significantly higher among all AD diagnostic subcohorts versus NPC smokers, with smokers with GAD reporting the highest levels of anxiety. The HADS depression scores were higher in smokers with GAD and PTSD than in those with PD and NPC.

**Table 1:** Baseline characteristics of all randomized participants by cohort

	AD subcohorts^a^ (*N* = 712)			NPC (*N* = 4028)	*p* value for significant difference (baseline variable by any cohort)^b^
	PTSD (*n* = 192)	GAD (*n* = 243)	PD (*n* = 277)		
Demographic characteristics					
Female, n (%)	107 (55.7)	149 (61.3)	183 (66.1)	2006 (49.8)	<.0001
Age (year), mean (SD)	45.1 (12.0)	47.5 (11.6)	44.5 (11.7)	45.9 (12.9)	.0465 (GAD > PD)
Race, *n* (%)					.0003
White	156 (81.3)	203 (83.5)	258 (93.1)	3324 (82.5)	
Black	23 (12.0)	33 (13.6)	14 (5.1)	514 (12.8)	
Other	12 (6.3)	7 (2.9)	5 (1.8)	190 (4.7)	
Region, *n* (%)					.0024
US	115 (59.9)	150 (61.7)	98 (35.4)	1901 (47.2)	
Non‐US^c^	77 (40.1)	93 (38.3)	179 (64.6)	2127 (52.8)	
BMI (kg/m^2^), mean (*SD*)	28.1 (7.0)	28.1 (6.4)	27.1 (5.8)	27.6 (6.1)	NS
Smoking characteristics					
FTCD score, mean (*SD*)	5.8 (2.1)	5.9 (2.0)	6.1 (2.1)	5.5 (2.0)	<.0001 (PD > NPC)
Duration of smoking (year), mean (*SD*)	26.8 (12.4)	28.5 (11.6)	26.9 (11.3)	28.1 (12.8)	NS
Cigarettes smoked per day in past month (*n*), mean (*SD*)	21.0 (8.9)	21.0 (7.9)	21.4 (7.7)	20.7 (8.0)	NS
Previous quit attempts (*n*), mean (*SD*)	3.6 (6.2)	3.1 (5.3)	2.4 (3.7)	3.2 (9.7)	NS
Prior use of study treatments					
Varenicline, *n* (%)	26 (13.5)	40 (16.5)	48 (17.3)	578 (14.3)	NS
Bupropion^d^, *n* (%)	21 (10.9)	27 (11.1)	26 (9.4)	373 (9.3)	NS
NRT, *n* (%)	48 (25.0)	67 (27.6)	70 (25.3)	998 (24.8)	NS
Psychiatric characteristics					
Any comorbid Axis I diagnosis, *n* (%)	100 (52.1)	107 (44.0)	93 (33.6)	15 (0.4)	<.0001
Substance‐use disorder history, *n* (%)	53 (27.6)	57 (23.5)	41 (14.8)	8 (0.2)	<.0001
Alcohol‐use disorder history	39 (20.3)	43 (17.7)	30 (10.8)	6 (0.1)	
Lifetime suicide‐related history from C‐SSRS, *n* (%)	66 (34.9)	46 (19.2)	43 (15.7)	194 (4.9)	<.0001
Suicidal ideation	66 (34.9)	46 (19.2)	42 (15.3)	190 (4.8)	
Suicidal behavior	23 (12.2)	10 (4.2)	6 (2.2)	28 (0.7)	
HADS score, mean (*SD*)					
Anxiety subscale score	5.6 (3.8)	6.6 (4.0)	4.9 (3.8)	2.8 (2.7)	<.0001 (GAD > PTSD > PD > NPC)
Depression subscale score	3.2 (3.4)	3.3 (3.1)	2.3 (2.9)	1.5 (2.1)	<.0001 (GAD, PTSD > PD > NPC)
BPAQ score, mean (*SD*)	59.8 (18.0)	59.1 (18.2)	57.5 (17.4)	52.2 (15.4)	<.0001 (PTSD, GAD, PD > NPC)
Receiving psychotropic medication at enrolment, *n* (%)	53 (27.6)	122 (50.2)	146 (52.7)	329 (8.2)	<.0001
Antidepressants	29 (15.1)	73 (30.0)	108 (39.0)	105 (2.6)	
Anxiolytics, hypnotics, and other sedatives	29 (15.1)	63 (25.9)	62 (22.4)	225 (5.6)	
Antipsychotics	12 (6.3)	6 (2.5)	10 (3.6)	13 (0.3)	
Mood stabilizers	0 (0.0)	4 (1.6)	2 (0.7)	20 (0.5)	
Other^e^	0 (0.0)	2 (0.8)	0 (0.0)	6 (0.1)	

Abbreviations: AD, anxiety disorder; BMI, body mass index; BPAQ, Buss–Perry Aggression Questionnaire; C‐SSRS, Columbia Suicide Severity Rating Scale; FTCD, Fagerström Test for Cigarette Dependence; GAD, generalized anxiety disorder; HADS, Hospital Anxiety and Depression Scale; NPC, nonpsychiatric cohort; NRT, nicotine‐replacement therapy (i.e., transdermal nicotine patch); NS, not significant; PD, panic disorder (with/without agoraphobia); PTSD, posttraumatic stress disorder; *SD*, standard deviation.

^a^ 80 participants from the AD subcohort with social phobia disorders (*n* = 48), obsessive‐compulsive disorders (*n* = 27), and without psychiatric disorders (*n* = 5) were excluded from the present analyses.

^b^ *p* value for second‐level comparison of baseline variable by any cohort (i.e., PTSD, GAD, PD, or NPC).

^c^ Argentina, Australia, Brazil, Bulgaria, Denmark, Canada, Chile, Finland, Germany, Mexico, New Zealand, Russia, Slovakia, South Africa, and Spain.

^d^ Bupropion prior use for smoking cessation or other indications.

^e^ Psychostimulants, amino acids, and herbals or botanicals.

### 3.2 Neuropsychiatric AEs

Table 2 illustrates the incidence of moderate to severe treatment‐emergent NPSAEs in each subcohort, the incidence of each individual component comprising the composite endpoint, and other prespecified NPSAE safety endpoints (e.g., serious NPSAEs). There was a significant AD subcohort effect (*p* = .0012), with smokers in all three AD subcohorts (PTSD, 6.9%; GAD, 5.4%; PD, 6.2%) having an increased incidence of moderate‐to‐severe NPSAEs versus NPC smokers (2.1%). Across the AD subcohorts, the rates of treatment discontinuation due to NPSAEs were similar, ranging from 1.3% (GAD) to 2.1% (PTSD).

**Table 2:** Incidence of moderate and severe treatment‐emergent^a^ neuropsychiatric adverse events (primary endpoint) among treated participants collapsed across treatment arms

	AD subcohorts (*N* = 703)			NPC (*N* = 3,984)	Comparative *p* value^b^
	PTSD (*n* = 189)	GAD (*n* = 240)	PD (*n* = 274)		
Observed incidence of the primary composite NPSAE endpoint^c^, *n* (%)	13 (6.9)	13 (5.4)	17 (6.2)	84 (2.1)	<.0001
Estimated primary composite NPSAE endpoint, % (95% CI)	5.22 (1.58, 8.85)	4.74 (1.80, 7.68)	5.42 (2.63, 8.22)	1.43 (0.70, 2.16)	
Observed components of primary composite NPSAE endpoint ≥1% in any cohort, *n* (%)					
Anxiety	1 (0.5)	3 (1.3)	2 (0.7)	4 (0.1)	.0003
Depression	3 (1.6)	0 (0.0)	1 (0.4)	1 (<0.1)	<.0001
Agitation	7 (3.7)	6 (2.5)	3 (1.1)	51 (1.3)	.0209
Aggression	1 (0.5)	4 (1.7)	5 (1.8)	11 (0.3)	.0001
Panic	2 (1.1)	2 (0.8)	7 (2.6)	8 (0.2)	<.0001
Observed events in the primary composite NPSAE endpoint of severe intensity only, *n* (%)	3 (1.6)	6 (2.5)	3 (1.1)	13 (0.3)	<.0001
Observed components of primary composite NPSAE endpoint of severe intensity only and ≥1% in any cohort, *n* (%)					
Anxiety	1 (0.5)	3 (1.3)	2 (0.7)	4 (0.1)	.0003
Depression	3 (1.6)	0 (0.0)	1 (0.4)	1 (<0.1)	<.0001
Events in the primary endpoint, *n* (%)					
Serious adverse events	0 (0.0)	1 (0.4)	0 (0.0)	6 (0.2)	.6056
Resulting in permanent treatment discontinuations	4 (2.1)	3 (1.3)	4 (1.5)	17 (0.4)	.0024
Combined serious adverse events, severe adverse events, and leading to treatment discontinuations or interventions (at least one of)	5 (2.6)	6 (2.5)	5 (1.8)	27 (0.7)	.0005
C‐SSRS‐ascertained suicidal ideation and/or behavior, *n* (%)	20 (10.6)	6 (2.6)	3 (1.1)	27 (0.7)	<.0001
Ideation	20 (10.6)	6 (2.6)	3 (1.1)	26 (0.7)	<.0001
Behavior	2 (1.1)	0 (0.0)	0 (0.0)	3 (0.1)	.0007

Abbreviations: AD, anxiety disorder; CI, confidence interval; C‐SSRS, Columbia Suicide Severity Rating Scale; GAD, generalized anxiety disorder; NPC, nonpsychiatric cohort; NPSAE, neuropsychiatric adverse event; PD, panic disorder (with/without agoraphobia); PTSD, posttraumatic stress disorder.

^a^ Occurring any time during 12 weeks of treatment and ≤30 days after the last dose.

^b^ Comparative *p* values are via the *χ* ^2^ test, not adjusted for multiplicity, and intended for descriptive purposes only.

^c^ *p* = .0012 for AD subcohorts versus NPC.

**Figure 1:**
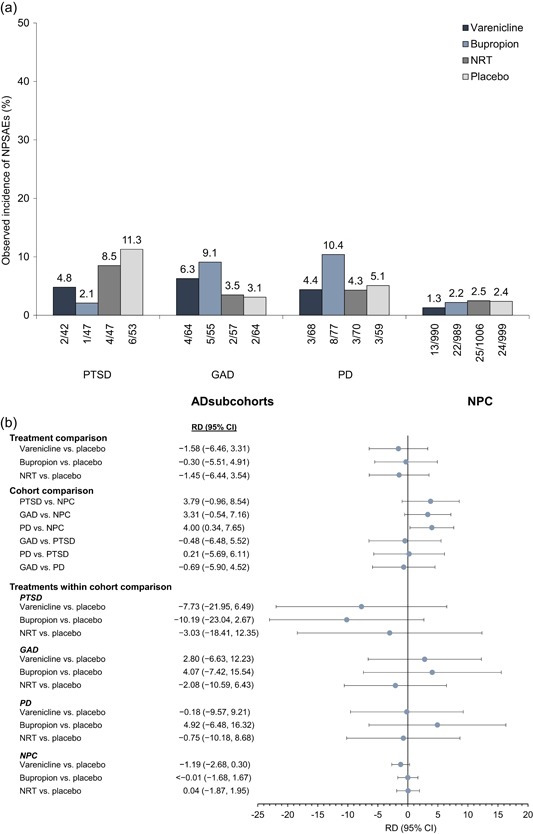
Incidence of neuropsychiatric adverse events (a) and risk differences (b) in the anxiety disorder subcohorts versus nonpsychiatric cohort. Period for the ascertainment of neuropsychiatric adverse events is during 12 weeks of treatment and ≤30 days after the last dose. The following variables were included in the risk‐difference model: treatment group, anxiety disorder subcohort, treatment‐by‐subcohort interaction, and region (US or non‐US). AD, anxiety disorder; CI, confidence interval; GAD, generalized anxiety disorder; NPC, nonpsychiatric cohort; NPSAE, neuropsychiatric adverse event; NRT, nicotine replacement therapy (i.e., transdermal nicotine patch); PD, panic disorder (with/without agoraphobia); PTSD, posttraumatic stress disorder; RD, risk difference

Figure 1a displays the observed incidence of the primary composite safety endpoint for each subcohort as a function of treatment. Figure 1b depicts risk differences (RDs) and associated 95% confidence intervals (95% CIs) for contrasts comparing: (a) treatments overall; (b) subcohorts; and (c) treatments within each subcohort and the NPC. There were no significant differences in the incidence of moderate to severe NPSAEs by treatment group, nor were there any significant treatment‐by‐cohort interactions. Smokers with PD were significantly more prone to experience moderate to severe NPSAEs versus NPC smokers (RD = 4.0; 95% CI = 0.34–7.65).

### 3.3 General AEs

Table 3 depicts 16 categories of psychosomatic AEs of any intensity occurring in ≥5% of participants in any subcohort. Sleep disorders and disturbances occurred at the highest rate (GAD, 26.7%). Gastrointestinal AEs were the second‐most commonly reported AE category (PTSD, 22.8%). Infections were the third‐most commonly reported AE category (NPC, 21.4%), followed by anxiety‐related AEs (PD, 19.0%) and headaches (PTSD, 15.9%). Interestingly, smokers with PTSD endorsed the highest rates of AEs among the subcohorts for 11 of 16 categories.

**Table 3:** Mild, moderate, or severe treatment‐emergent adverse events^a^ reported by ≥5% of participants in any subcohort

Adverse event, *n* (%)	AD subcohorts (*N* = 703)			NPC (*n* = 3,984)	Comparative *p* value^b^
	PTSD (*n* = 189)	GAD (*n* = 240)	PD (*n* = 274)		
Gastrointestinal motility and defecation conditions	15 (7.9)	9 (3.8)	22 (8.0)	252 (6.3)	.1850
Gastrointestinal signs and symptoms	43 (22.8)	40 (16.7)	60 (21.9)	697 (17.5)	.0824
Administration‐site reactions	11 (5.8)	8 (3.3)	10 (3.6)	180 (4.5)	.5746
General system disorders NEC	21 (11.1)	16 (6.7)	28 (10.2)	333 (8.4)	.2792
Infections—pathogen unspecified	35 (18.5)	41 (17.1)	56 (20.4)	852 (21.4)	.3434
Viral infectious disorders	11 (5.8)	8 (3.3)	13 (4.7)	152 (3.8)	.4509
Injuries NEC	15 (7.9)	8 (3.3)	18 (6.6)	0 (0.0)	<.0001
Joint disorders	10 (5.3)	5 (2.1)	6 (2.2)	75 (1.9)	.0149
Headaches	30 (15.9)	22 (9.2)	30 (10.9)	447 (11.2)	.1648
Neurological disorders NEC	21 (11.1)	31 (12.9)	39 (14.2)	317 (8.0)	.0002
Anxiety disorders and symptoms	32 (16.9)	40 (16.7)	52 (19.0)	369 (9.3)	<.0001
Depressed‐mood disorders and disturbances	21 (11.1)	15 (6.3)	22 (8.0)	177 (4.4)	<.0001
Mood disorders and disturbances NEC	20 (10.6)	21 (8.8)	19 (6.9)	201 (5.0)	.0008
Sleep disorders and disturbances	42 (22.2)	64 (26.7)	64 (23.4)	787 (19.8)	.0338
Respiratory disorders NEC	15 (7.9)	7 (2.9)	10 (3.6)	219 (5.5)	.0709
Epidermal and dermal conditions	17 (9.0)	13 (5.4)	12 (4.4)	186 (4.7)	.0552

Abbreviations: AD, anxiety disorder; GAD, generalized anxiety disorder; NEC, not elsewhere classified; NPC, nonpsychiatric cohort; PD, panic disorder (with/without agoraphobia); PTSD, posttraumatic stress disorder.

^a^ As classified by the Medical Dictionary for Regulatory Activities (MedDRA, v18.0) high‐level group terms, and occurring any time during 12 weeks of treatment and ≤30 days after the last dose.

^b^ Comparative *p* values are via the *χ* ^2^ test, not adjusted for multiplicity, and intended for descriptive purposes only.

### 3.4 Efficacy

**Figure 2:**
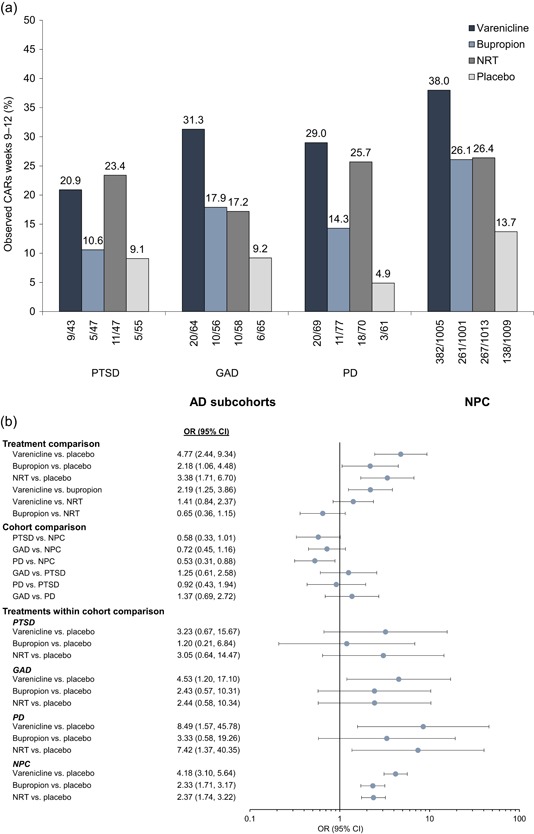
Observed continuous abstinence rates (a) and odds ratios (b) for weeks 9–12. The following variables were included in the odds‐ratio model: treatment group, anxiety disorder subcohort, treatment‐by‐subcohort interaction, region (US or non‐US), race, age, Fagerström Test for Cigarette Dependence, and cigarettes smoked/day in the past month. AD, anxiety disorder; CAR, continuous abstinence rate; CI, confidence interval; GAD, generalized anxiety disorder; NPC, nonpsychiatric cohort; NRT, nicotine replacement therapy (i.e., transdermal nicotine patch); OR, odds ratio; PD, panic disorder (with/without agoraphobia); PTSD, posttraumatic stress disorder

Figure 2a shows the primary efficacy endpoint (CAR9–12) across subcohorts as a function of treatment, while Figure 2b provides effect size estimates for (a) treatments overall; (b) subcohorts; and (c) treatments within each subcohort and the NPC. Smokers with PD were significantly less likely to quit than NPC smokers (odds ratio [OR] = 0.53; 95% CI = 0.31–0.88). Among smokers with PTSD, there were no significant differences in CAR9–12 for any active treatment versus placebo; however, ORs for varenicline and NRT versus placebo were notably >3. Among smokers with GAD and PD, varenicline demonstrated superior efficacy to placebo (OR = 4.53; 95% CI = 1.20–17.10; and OR = 8.49; 95% CI = 1.57–45.78, respectively). Additionally, among smokers with PD, NRT significantly improved quit rates versus placebo (OR = 7.42; 95% CI = 1.37–40.35). Thus, although we found no frank interaction between primary AD diagnosis and treatment response, there appears to be beneficial effects on initiating smoking abstinence for varenicline and NRT in smokers with AD.

The secondary efficacy endpoint, weekly PPA at weeks 12 and 24, are presented in Figure 3. Consistent with some of the CAR9–12 results, these suggest the possibility of differential treatment responses to the first‐line smoking cessation medications by primary AD diagnosis. For example, varenicline showed superior efficacy to placebo on 7‐day PPA at week 12 in smokers with PTSD, GAD, and PD. This effect remained at week 24 among smokers with GAD. In addition, varenicline was superior to bupropion and NRT at week 12 among smokers with GAD. Among smokers with PD, NRT was superior to placebo for 7‐day PPA at both weeks 12 and 24.

Figure 3Observed 7‐day point prevalence of abstinence during treatment and follow‐up and odds ratios at week 12 and week 24 by cohort. The following variables were included in the odds‐ratio model: treatment group, anxiety disorder subcohort, treatment‐by‐subcohort interaction, and region (US or non‐US) and region‐by‐cohort interaction. CI, confidence interval; GAD, generalized anxiety disorder; NPC, nonpsychiatric cohort; NRT, nicotine replacement therapy (i.e., transdermal nicotine patch); OR, odds ratio; PD, panic disorder (with/without agoraphobia); PPA, point prevalence of abstinence; PTSD, posttraumatic stress disorder

**Figure:**
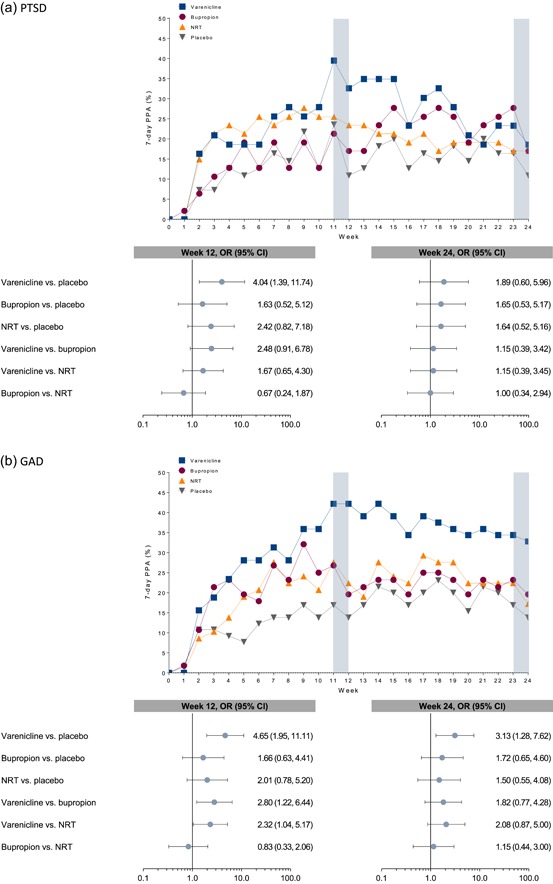

**Figure:**
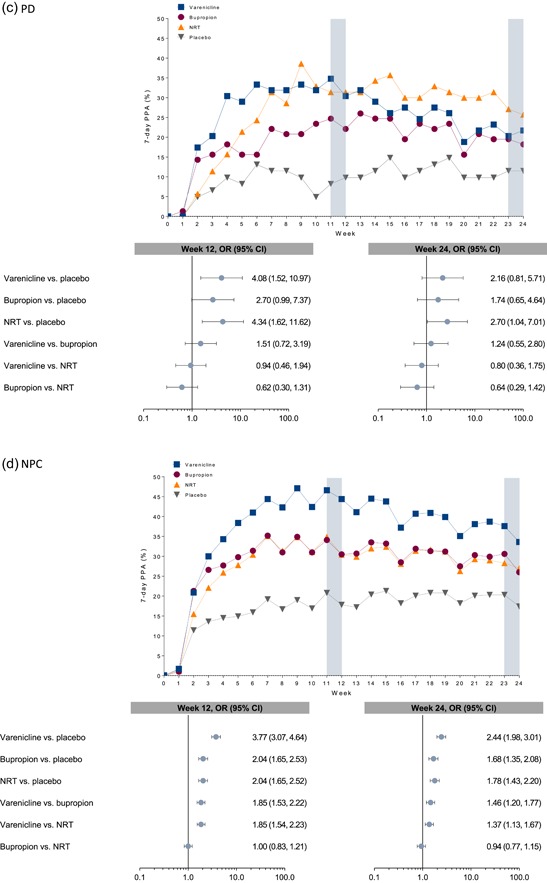

Observed CAR9–24 for varenicline, bupropion, NRT, and placebo ranged from 7 to 13% (PTSD), 6–22% (GAD), 5–21% (PD), and 11–26% (NPC), with placebo consistently the lowest (Figure S2). Within AD subcohorts, estimated ORs versus placebo for CAR9–24 were >2 for all active treatments, with the exception of bupropion and varenicline in smokers with PTSD. However, the 95% CIs for all comparisons included one, with the exception of NRT in smokers with PD, which continued to demonstrate longer‐term efficacy in that subcohort.

## 4 DISCUSSION

Our results from the largest, prospective, placebo‐controlled RCT of smoking cessation medications in smokers with AD provide evidence that varenicline, bupropion, and NRT are generally well tolerated in smokers with PTSD, GAD, and PD, and that varenicline and NRT aid quitting in these diagnostic subgroups, albeit at rates lower than in smokers without mental health conditions.

Like smokers with other mental health conditions (Anthenelli et al., 2016; Evins et al., 2019), smokers with primary diagnoses of PTSD, GAD, and PD with/without agoraphobia were each more likely to experience moderate to severe NPSAEs during a quit attempt than smokers without mental health conditions, regardless of treatment. This finding is not surprising given the overlap of ADs and heightened anxiety sensitivity (Abrams et al., 2017) and distress intolerance (Farris et al., 2016aa). Although the psychiatric NPSAE events that comprised the primary safety outcome had to be severe enough to interfere significantly with functioning, it is possible that hyperarousal to symptoms in smokers with AD contributed to a greater occurrence of NPSAEs. We also found that compared with smokers without mental health conditions, diagnostic subgroups of smokers with AD had higher baseline levels of state anxiety, histories of suicidal ideation/behavior, and were more likely to be white females—variables we have previously reported were independently associated with heightened NPSAE risk (Anthenelli et al., 2019). However, our data on the incidence of these events in subtypes of smokers with AD provides some reassurance that they occur relatively infrequently and are not related to any specific pharmacotherapy. Of particular interest given the prior concerns about the use of varenicline in smokers with PTSD (Campbell & Anderson, 2010; Cunningham et al., 2016) is that these events occurred most commonly with placebo and were lowest with the two non‐nicotine medications (Figure 1a).

Based on CAR9–12 outcomes and the 95% CIs overlapping one, it would appear at first glance that smokers with PTSD might not respond to any of the three first‐line medications. However, closer inspection of the ORs (>3 for both varenicline and NRT vs. placebo) for the CAR9–12 data and the significant effect (OR = 4.04; 95% CI = 1.39–11.74) for varenicline versus placebo for 7‐day PPA at week 12 (Figure 3) might make such an interpretation premature. Thus, we conclude that while overall smokers with PTSD had 42% lower odds of quitting versus smokers without mental health conditions, varenicline and NRT hold promise in these difficult‐to‐treat smokers, while there is less of a signal for bupropion. However, larger head‐to‐head studies are needed to test those hypotheses because some studies have found beneficial effects for bupropion in smokers with PTSD (Hertzberg, Moore, Feldman, & Beckham, 2001; McFall et al., 2010). Our finding is consistent with a retrospective, open‐label trial of varenicline (Okoli, Wiggins, Fallin‐Bennett, & Rayens, 2017), and a concurrent varenicline and prolonged‐exposure therapy trial in smokers with PTSD (Foa et al., 2017), both of which showed beneficial effects of varenicline in smokers with PTSD. They are also consistent with nonplacebo‐controlled trials of NRT (Hertzberg et al., 2013; Kelly et al., 2015aa; Kelly et al., 2015bb; McFall et al., 2010) showing beneficial effects in smokers with PTSD.

Among smokers with GAD, we observed a pattern of results similar to that found in smokers with heterogeneous mental health conditions that we described in our initial report (Anthenelli et al., 2016): odds of quitting were highest for varenicline versus other active treatments. However, in contrast to those results, and likely due to the limited power in the GAD subcohort, the effects for bupropion (OR = 2.43; 95% CI = 0.57–10.31) and for NRT (OR= 2.44; 95% CI = 0.58–10.34) versus placebo did not reach statistical significance in these smaller samples. Nonetheless, we conclude that all three medications might show efficacy in smokers with GAD, albeit with varenicline having a larger effect based on the significant 7‐day PPA at week 12 comparisons.

Smokers with PD with/without agoraphobia appeared to respond similarly to NRT and varenicline. In fact, for CAR9–12 as well as 7‐day PPA at week 12, both medications were superior to placebo. This equivalent pattern of response, while similar to that for CAR9–12 in smokers with PTSD, is the first we have observed among any psychiatric subcohort of smokers we have studied to date in EAGLES, who typically show the pattern we described previously, wherein varenicline is better than bupropion and NRT, and all are better than placebo (Anthenelli et al., 2016). This novel finding warrants replication, but it is interesting to note that varenicline and NRT partially or fully agonize nicotinic acetylcholine receptors and ameliorate nicotine withdrawal, which has been found to be more severe in certain smokers with AD prone to negative reinforcement‐based smoking (Farris et al., 2016bb; Leventhal & Zvolensky, 2015).

To our knowledge, there has been only one other placebo‐controlled trial of bupropion, NRT, and combinations thereof in smokers with AD to date. Piper and colleagues (Piper et al., 2011) retrospectively examined the relationship between ADs (including panic attack, social anxiety disorder, and GAD) and reported that neither bupropion, NRT, nor their combination was more effective than placebo and counseling. Our group found some differences among smokers with AD in that varenicline provided significant benefit for cessation in those with GAD and PD, while NRT provided significant benefit for those with PD. The discrepancy with our results and Piper's investigation may have had to do with that group's smaller sample size, use of a structured interview that included lifetime diagnoses, and diagnostic differences (e.g., history of panic attacks vs. PD). Additional investigations (Gaspersz et al., 2017) examined anxiety in depressed patients and suggested that smokers with comorbid AD may be less able to deal with the stress and challenges of smoking cessation. While our results supported the idea that smokers with AD have lower rates of abstinence and higher rates of AEs during a cessation attempt than smokers with no psychiatric disorder, we also present evidence that first‐line smoking cessation treatments do indeed work in those with ADs. ORs for CAR9–12 for active treatments versus placebo ranged from 1.20 to 3.23 (PTSD), 2.43–4.53 (GAD), and 3.33–8.49 (PD). Thus, clinicians should utilize these treatments, as they produce clinical gains and do not increase the risk of moderate to severe NPSAEs, which occurred at low frequency overall and with no differences between treatment groups. Of note, there was lower efficacy of smoking cessation treatment on longer‐term (CAR9–24) outcomes indicating smokers with ADs may require prolonged pharmacotherapy use or continued smoking cessation counseling.

The primary limitations of this study are related to generalizability. This study did not include individuals who were psychiatrically unstable or those with current substance‐use disorders. The most recent DSM‐5 no longer categorizes PTSD as an AD, but rather, a trauma‐related disorder; nonetheless, there is substantial overlap in the symptoms of PTSD and ADs. Small samples of those with social phobia disorder and obsessive‐compulsive disorder limited our ability to evaluate these diagnostic groups. Given the post hoc nature of this study, we did not adjust for multiple comparisons, which increases the probability of a Type I error. Finally, this study was not powered or stratified a priori for this subcohort analysis.

In conclusion, individuals with primary PTSD, GAD, and PD were approximately three times more likely to experience clinically significant NPSAEs during medication‐assisted cessation attempts and were 28% (GAD) to 47% (PD) less likely to quit than smokers without mental health conditions. The incidence of moderate to severe NPSAEs was similar across the three AD subcohorts and not related to any specific smoking cessation treatment. Despite limited power in some diagnostic subcohorts, compared with placebo and counselling, varenicline boosted odds of quitting threefold in smokers with PTSD (95% CI = 0.67–15.67), 4.5‐fold in smokers with GAD (95% CI = 1.20–17.10), and 8.5‐fold in PD smokers (95% CI = 1.57–45.78). Additionally, among PD smokers, NRT improved quit rates approximately 7.5‐fold versus placebo (95% CI = 1.37–40.35) while also tripling the quit rate in smokers with PTSD (95% CI = 0.64–14.47). Our results demonstrate that the first‐line (varenicline, bupropion, NRT) smoking cessation treatments are effective at least in the short‐term for smokers with PTSD, GAD, and PD. Smokers with AD, particularly those with PTSD, are not all alike with respect to their baseline smoking and psychiatric characteristics, AE patterns, and treatment response. Differences between distinct anxiety and trauma‐related disorders should be further explored. Regardless of whether there are larger clinical trials in the future, smoking cessation efforts utilizing these first‐line treatments should be considered for smokers with ADs.

## CONFLICT OF INTERESTS

C. R. A. and J. L. H. report no conflicts of interest. A. E. E. reports research grants to her institution from Forum Pharmaceuticals and Pfizer and personal fees for consultation or advisory board services from Charles River Analytics, Karuna Pharmaceuticals, Alkermes, Pfizer, and Reckitt Benck iser. A. E. E.'s writing of the manuscript was supported by a National Institute on Drug Abuse Career Award in Patient‐Oriented Research, K24 DA030443. R. M. A. reports his university receiving grants from Alkermes and Pfizer and providing consulting and/or advisory board services to Arena Pharmaceuticals, Cerecor, Pfizer, and US WorldMeds. R. M. A.'s writing of this manuscript was supported, in part, by National Institute on Alcohol Abuse and Alcoholism Grant #s U10 AA008401 and 1R44AA024643, and NIDA Grant # UO1 DA041731. C. R., D. L., and T. M. are employees and stockholders of Pfizer. The opinions expressed in this article are those of the authors and do not necessarily reflect the views of their employers.

## AUTHOR CONTRIBUTIONS

All authors, C. R. A., J. L. H., C. R., D. L., T. M., A. E. E., and R. M. A., were involved in the analyses and/or interpretation of data; D. L. performed the statistical analyses; C. R. A. drafted the initial manuscript; and all authors have critically revised the manuscript for content and have approved the final version and the decision to submit for publication.

## Supporting information

Supporting information

## Data Availability

Upon request, and subject to certain criteria, conditions, and exceptions (see https://www.pfizer.com/science/clinical-trials/trial-data-and-results for more information), Pfizer will provide access to individual deidentified participant data from Pfizer‐sponsored global interventional clinical studies conducted for medicines, vaccines, and medical devices: (a) for indications that have been approved in the USA and/or EU; or (b) in programs that have been terminated (i.e., development for all indications has been discontinued). Pfizer will also consider requests for the protocol, data dictionary, and statistical analysis plan. Data may be requested from Pfizer trials 24 months after study completion. The deidentified participant data will be made available to researchers whose proposals meet the research criteria and other conditions, and for which an exception does not apply, via a secure portal. To gain access, data requestors must enter into a data access agreement with Pfizer. Data subject to third party restrictions.

## References

[da22982-bib-0001] Abrams, K. , Krimmel, S. , Johnson, S. , Cieslowski, K. , Strnad, H. , Melum, A. , & Kryder, C. (2017). Nicotine deprivation attenuates panic reactivity in smokers: Findings from a placebo‐controlled nicotine patch study. Depression and Anxiety, 34(11), 996–1005. 10.1002/da.22652 28489321

[da22982-bib-0002] American Psychiatric Association . (2000). Diagnostic and statistical manual of mental disorders (4th ed.). Arlington, VA: American Psychiatric Association.

[da22982-bib-0003] Anthenelli, R. M. , Benowitz, N. L. , West, R., St , Aubin, L. , McRae, T. , Lawrence, D. , … Evins, A. E. (2016). Neuropsychiatric safety and efficacy of varenicline, bupropion, and nicotine patch in smokers with and without psychiatric disorders (EAGLES): A double‐blind, randomised, placebo‐controlled clinical trial. Lancet, 387(10037), 2507–2520. 10.1016/S0140-6736(16)30272-0 27116918

[da22982-bib-0004] Anthenelli, R. M. , Gaffney, M. , Benowitz, N. L. , West, R. , McRae, T. , Russ, C. , … Evins, A. E. (2019). Predictors of neuropsychiatric adverse events with smoking cessation medications in the randomized controlled EAGLES trial. Journal of General Internal Medicine, 34, 862–870. 10.1007/s11606-019-04858-2 30847828 PMC6544691

[da22982-bib-0005] Anthenelli, R. M. , Morris, C. , Ramey, T. S. , Dubrava, S. J. , Tsilkos, K. , Russ, C. , & Yunis, C. (2013). Effects of varenicline on smoking cessation in adults with stably treated current or past major depression: A randomized trial. Annals of Internal Medicine, 159(6), 390–400. 10.7326/0003-4819-159-6-201309170-00005 24042367

[da22982-bib-0006] Benowitz, N. L. , Jacob, P., III , Ahijevych, K. , Jarvis, M. J. , Hall, S. , & LeHouezec, J. , (SRNT Subcommittee on Biochemical Verification) . (2002). Biochemical verification of tobacco use and cessation. Nicotine & Tobacco Research, 4(2), 149–159. 12028847 10.1080/14622200210123581

[da22982-bib-0007] Buss, A. H. , & Perry, M. (1992). The aggression questionnaire. Journal of Personality and Social Psychology, 63(3), 452–459. 1403624 10.1037//0022-3514.63.3.452

[da22982-bib-0008] Campbell, A. R. , & Anderson, K. D. (2010). Mental health stability in veterans with posttraumatic stress disorder receiving varenicline. American Journal of Health‐System Pharmacy, 67(21), 1832–1837. 10.2146/ajhp100196 20966147

[da22982-bib-0009] Cougle, J. R. , Zvolensky, M. J. , Fitch, K. E. , & Sachs‐Ericsson, N. (2010). The role of comorbidity in explaining the associations between anxiety disorders and smoking. Nicotine & Tobacco Research, 12(4), 355–364. 10.1093/ntr/ntq006 20156885 PMC2847074

[da22982-bib-0010] Cunningham, F. E. , Hur, K. , Dong, D. , Miller, D. R. , Zhang, R. , Wei, X. , … Good, C. B. (2016). A comparison of neuropsychiatric adverse events during early treatment with varenicline or a nicotine patch. Addiction, 111(7), 1283–1292. 10.1111/add.13329 26826702

[da22982-bib-0011] Evins, A. E. , Benowitz, N. L. , West, R. , Russ, C. , McRae, T. , Lawrence, D. , … Anthenelli, R. M. (2019). Neuropsychiatric safety and efficacy of varenicline, bupropion, and nicotine patch in smokers with psychotic, anxiety, and mood disorders in the EAGLES trial. Journal of Clinical Psychopharmacology, 39(2), 108–116. 10.1097/JCP.0000000000001015 30811371 PMC6488024

[da22982-bib-0012] Evins, A. E. , Cather, C. , & Laffer, A. (2015). Treatment of tobacco use disorders in smokers with serious mental illness: Toward clinical best practices. Harvard Review of Psychiatry, 23(2), 90–98. 10.1097/HRP.0000000000000063 25747922 PMC4460830

[da22982-bib-0013] Fagerström, K. (2012). Determinants of tobacco use and renaming the FTND to the Fagerstrom Test for Cigarette Dependence. Nicotine & Tobacco Research, 14(1), 75–78. 10.1093/ntr/ntr137 22025545

[da22982-bib-0014] Farris, S. G. , Leyro, T. M. , Allan, N. P. , Overup, C. S. , Schmidt, N. B. , & Zvolensky, M. J. (2016a). Distress intolerance during smoking cessation treatment. Behaviour Research and Therapy, 85, 33–42. 10.1016/j.brat.2016.08.002 27565398 PMC5026956

[da22982-bib-0015] Farris, S. G. , Robinson, J. D. , Zvolensky, M. J. , Hogan, J. , Rabius, V. , Cinciripini, P. M. , … Blalock, J. A. (2016b). Panic attacks and smoking cessation among cancer patients receiving smoking cessation treatment. Addictive Behaviors, 61, 32–39. 10.1016/j.addbeh.2016.05.011 27235990 PMC5912332

[da22982-bib-0016] Federal Aviation Administration . (2008). What do physicians know about normal pressure hydrocephalus and when did they know it? A survey of 284 physicians, Anti‐Smoking Medicine Chantix Banned, 81, 19–29. https://www.faa.gov/news/updates/?newsId=56363 PMC2442723 18604308

[da22982-bib-0017] Fiore, M. C. , Jaén, C. R. , Baker, T. B. , Bailey, W. C. , Benowitz, N. L. , Curry, S. J. , … Wewers, M. E. (2008). Treating Tobacco Use and Dependence: 2008 Update. In Clinical Practice Guideline. Rockville. Maryland: Department of Health and Human Services. Public Health Service.

[da22982-bib-0018] First, M. B. , Gibbon, M. , Spitzer, R. L. , Williams, J. B. W. , & Benjamin, L. S. (1997). User's Guide for the Structured Clinical Interview for DSM‐IV Axis II Personality Disorders: SCID‐II (pp. 1–41). American Psychiatric Press.

[da22982-bib-0019] First, M. B. , Spitzer, R. L. , Gibbon, M. , & Williams, J. B. W. (2002). Structured Clinical Interview for DSM‐IV‐TR Axis I Disorders, Research Version, Non‐patient Edition (SCID‐I/NP). New York: Biometrics Research, New York State Psychiatric Institute.

[da22982-bib-0020] Foa, E. B. , Asnaani, A. , Rosenfield, D. , Zandberg, L. J. , Gariti, P. , & Imms, P. (2017). Concurrent varenicline and prolonged exposure for patients with nicotine dependence and PTSD: A randomized controlled trial. Journal of Consulting and Clinical Psychology, 85(9), 862–872. 10.1037/ccp0000213 28569519 PMC5578908

[da22982-bib-0021] Gaspersz, R. , Lamers, F. , Kent, J. M. , Beekman, A. T. F. , Smit, J. H. , van Hemert, A. M. , … Penninx, B. (2017). Anxious distress predicts subsequent treatment outcome and side effects in depressed patients starting antidepressant treatment. Journal of Psychiatric Research, 84, 41–48. 10.1016/j.jpsychires.2016.09.018 27693981

[da22982-bib-0022] Hertzberg, J. S. , Carpenter, V. L. , Kirby, A. C. , Calhoun, P. S. , Moore, S. D. , Dennis, M. F. , … Beckham, J. C. (2013). Mobile contingency management as an adjunctive smoking cessation treatment for smokers with posttraumatic stress disorder. Nicotine & Tobacco Research, 15(11), 1934–1938. 10.1093/ntr/ntt060 23645606 PMC3790624

[da22982-bib-0023] Hertzberg, M. A. , Moore, S. D. , Feldman, M. E. , & Beckham, J. C. (2001). A preliminary study of bupropion sustained‐release for smoking cessation in patients with chronic posttraumatic stress disorder. Journal of Clinical Psychopharmacology, 21(1), 94–98. 11199956 10.1097/00004714-200102000-00017

[da22982-bib-0024] Kelly, M. M. , Jensen, K. P. , & Sofuoglu, M. (2015a). Co‐occurring tobacco use and posttraumatic stress disorder: Smoking cessation treatment implications. The American Journal on Addictions, 24(8), 695–704. 10.1111/ajad.12304 26584242

[da22982-bib-0025] Kelly, M. M. , Sido, H. , Forsyth, J. P. , Ziedonis, D. M. , Kalman, D. , & Cooney, J. L. (2015b). Acceptance and commitment therapy smoking cessation treatment for veterans with posttraumatic stress disorder: A pilot study. Journal of dual diagnosis, 11(1), 50–55. 10.1080/15504263.2014.992201 25491589

[da22982-bib-0026] Lasser, K. , Boyd, J. W. , Woolhandler, S. , Himmelstein, D. U. , McCormick, D. , & Bor, D. H. (2000). Smoking and mental illness: A population‐based prevalence study. Journal of the American Medical Association, 284(20), 2606–2610. 11086367 10.1001/jama.284.20.2606

[da22982-bib-0027] Leventhal, A. M. , & Zvolensky, M. J. (2015). Anxiety, depression, and cigarette smoking: A transdiagnostic vulnerability framework to understanding emotion‐smoking comorbidity. Psychological Bulletin, 141(1), 176–212. 10.1037/bul0000003 25365764 PMC4293352

[da22982-bib-0028] McCabe, R. E. , Chudzik, S. M. , Antony, M. M. , Young, L. , Swinson, R. P. , & Zolvensky, M. J. (2004). Smoking behaviors across anxiety disorders. Journal of Anxiety Disorders, 18(1), 7–18. 14725865 10.1016/j.janxdis.2003.07.003

[da22982-bib-0029] McFall, M. , Saxon, A. J. , Malte, C. A. , Chow, B. , Bailey, S. , Baker, D. G. , & Team, C. S. P. S. (2010). Integrating tobacco cessation into mental health care for posttraumatic stress disorder: A randomized controlled trial. Journal of the American Medical Association, 304(22), 2485–2493. 10.1001/jama.2010.1769 21139110 PMC4218733

[da22982-bib-0030] Okoli, C. T. C. , Otachi, J. K. , Manuel, A. , & Woods, M. (2018). A cross‐sectional analysis of factors associated with the intention to engage in tobacco treatment among inpatients in a state psychiatric hospital. Journal of Psychiatric and Mental Health Nursing, 25(1), 14–25. 10.1111/jpm.12435 28976063

[da22982-bib-0031] Okoli, C. T. C. , Wiggins, A. , Fallin‐Bennett, A. , & Rayens, M. K. (2017). A retrospective analysis of the comparative effectiveness of smoking cessation medication among individuals with mental illness in community‐based mental health and addictions treatment settings. Journal of Psychiatric and Mental Health Nursing, 24(8), 610–619. 10.1111/jpm.12408 28635015

[da22982-bib-0032] Piper, M. E. , Cook, J. W. , Schlam, T. R. , Jorenby, D. E. , & Baker, T. B. (2011). Anxiety diagnoses in smokers seeking cessation treatment: Relations with tobacco dependence, withdrawal, outcome, and response to treatment. Addiction, 106(2), 418–427. 10.1111/j.1360-0443.2010.03173.x 20973856 PMC3017215

[da22982-bib-0033] Posner, K. , Brown, G. K. , Stanley, B. , Brent, D. A. , Yershova, K. V. , Oquendo, M. A. , … Mann, J. J. (2011). The Columbia‐Suicide Severity Rating Scale: Initial validity and internal consistency findings from three multisite studies with adolescents and adults. American Journal of Psychiatry, 168(12), 1266–1277. 10.1176/appi.ajp.2011.10111704 22193671 PMC3893686

[da22982-bib-0034] US Department of Defence (2008). DoD Medication Safety Notice: Varenicline (Chantix). Retrieved from https://www.healthquality.va.gov/guidelines/CD/mtu/varenicline_safety_notice.pdf

[da22982-bib-0035] US Department of Veterans Affairs, VA Center for Medication Safety, Tobacco Use Cessation Technical Advisory Group, Public Health Strategic Healthcare Group, VA Pharmacy Benefits Management Services, VISN Pharmacist Executives, & Medical Advisory Panel (2008), July 2011). Varenicline criteria for prescribing. Retrieved from https://www.healthquality.va.gov/tuc/VareniclineCriteriaforPrescribing.pdf

[da22982-bib-0036] West, R. , Hajek, P. , Stead, L. , & Stapleton, J. (2005). Outcome criteria in smoking cessation trials: Proposal for a common standard. Addiction, 100(3), 299–303. 10.1111/j.1360-0443.2004.00995.x 15733243

[da22982-bib-0037] Zigmond, A. S. , & Snaith, R. P. (1983). The hospital anxiety and depression scale. Acta Psychiatrica Scandinavica, 67(6), 361–370. 6880820 10.1111/j.1600-0447.1983.tb09716.x

