# Supplementary material for: Efficacy and safety of pharmacotherapies for smoking cessation in anxiety disorders: Subgroup analysis of the randomized, active‐ and placebo‐controlled EAGLES trial
Source: Depress Anxiety. 2019 Dec 18;37(3):247–60. doi: 10.1002/da.22982 (PMC7064930; doi:10.1002/da.22982)
Supplement: Supplementary file 1 — Supporting information [file DA-37-247-s001.docx]

**Supporting Table T1** Final model summaries for neuropsychiatric adverse events, continuous abstinence for weeks 9–12 and weeks 9–24, and 7-day point prevalence abstinence at weeks 12 and 24 (covariate selection)

| **Term** | **NPSAEs** | **CA9-12** | **CA9-24** | **PPA Week 12** | **PPA Week 24** |
| --- | --- | --- | --- | --- | --- |
| Treatment^†^ | X | X | X | X | X |
| Cohort | X | X | X | X | X |
| Treatment*Cohort | X | X | X | X | X |
| Region | X | X | X | X | X |
| Cohort*Region |  | X | X | X | X |
|  |  |  |  |  |  |
| **Categorical** |  |  |  |  |  |
| Gender |  |  |  |  |  |
| Race (3 level) |  | 5 | 5 | 4 |  |
| Alcohol/substance-use disorder |  |  |  |  |  |
| Psychotropic medication |  | 7 |  | 7 |  |
| Suicidal ideation (C-SSRS) |  |  |  |  |  |
| Suicidal behavior (C-SSRS) |  |  |  |  |  |
| Suicidal ideation and/or behavior (C-SSRS) | 4 |  |  |  |  |
| Psychotropic medication (enrolment) |  |  |  |  |  |
| Antidepressants |  |  |  |  |  |
| Anxiolytics, hypnotics, and other sedatives |  |  | 10 |  |  |
| Antipsychotics |  |  |  |  |  |
| Mood stabilizers |  |  |  |  |  |
| Other |  |  |  |  |  |
| Prior varenicline |  |  |  |  |  |
| Prior bupropion |  |  |  |  |  |
| Prior NRT |  |  |  |  |  |
| Comorbid Axis I diagnosis |  |  |  |  |  |
| Lives with smoker |  |  |  |  |  |
| Contact with smoker |  | 2 | 2 | 3 | 3 |
| Number of lifetime serious quit attempt |  |  |  |  |  |
| Alcohol abuse disorder comorbidity |  |  |  |  |  |
| Substance abuse disorder comorbidity | 2 |  |  |  |  |
| CGI-S |  |  |  |  |  |
|  |  |  |  |  |  |
| **Numerical** |  |  |  |  |  |
| FTCD |  | 1 | 1 | 1 | 1 |
| Age |  | 4 | 7 | 6 |  |
| BMI |  |  | 4 | 5 | 5 |
| HADS anxiety subscale (baseline) | 1 |  |  |  |  |
| HADS depression subscale (baseline) |  |  |  |  |  |
| BPAQ |  |  |  |  |  |
| BPAQ_physical aggression | 3 |  |  |  |  |
| AQ_verbal aggression |  |  |  |  |  |
| AQ_anger |  |  |  |  |  |
| AQ_hostility |  |  |  |  |  |
| Weight |  |  |  |  |  |
| Start age of smoking | 5 |  | 3, −9 |  | 4 |
| Duration of smoking |  | 6 | 8 |  |  |
| Cigarettes per day in the past month |  | 3 | 6 | 2 | 2 |
| No medical comorbidity |  |  |  |  |  |
| No SCID comorbidity |  |  |  |  | 6, −7 |

BMI, body mass index; BPAQ, Buss–Perry Aggression Questionnaire; CA, continuous abstinence; CGI-S, Clinical Global Impression – Severity scale; C-SSRS, Columbia Suicide Severity Rating Scale; FTCD, Fagerström Test for Cigarette Dependence; HADS, Hospital Anxiety and Depression Scale; NPC, non-psychiatric cohort; NPSAE, neuropsychiatric adverse event; NRT, nicotine-replacement therapy (i.e., transdermal nicotine patch); PPA, point prevalence abstinence; SCID, Structured Clinical Interview for the Diagnostic and Statistical Manual of Mental Disorders, Fourth Edition, Text Revision (DSM-IV-TR) Axis-I/-II Disorders.

^†^ "X" indicates a forced inclusion term and a number identifies the inclusion (or removal, if negative) step for that term.

**Supporting Figure S1** Participant disposition

AD, anxiety disorder; GAD, generalized anxiety disorder; NPC, non-psychiatric cohort; PC, psychiatric cohort; PD, panic disorder (with/without agoraphobia); PTSD, post-traumatic stress disorder.

**Supporting Figure S2** Observed continuous abstinence rates (a) and odds ratios (b) for weeks 9–24. The following variables were included in the odds ratio model: Treatment group, anxiety disorder subcohort, treatment-by-subcohort interaction, region (US or non-US), race, age, body mass index, Fagerström Test for Cigarette Dependence, cigarettes smoked/day in the past month, duration of smoking, and prior use of NRT

AD, anxiety disorder; CAR, continuous abstinence rate; CI, confidence interval; GAD, generalized anxiety disorder; NPC, non-psychiatric cohort; NRT, nicotine replacement therapy (i.e., transdermal nicotine patch); OR, odds ratio; PD, panic disorder (with/without agoraphobia); PTSD, post-traumatic stress disorder.
